# Association of *Helicobacter pylori* Infection with Coronary Artery Disease: Is *Helicobacter pylori* a Risk Factor?

**DOI:** 10.1155/2014/516354

**Published:** 2014-01-16

**Authors:** Jamshid Vafaeimanesh, Seyyed Fakhroldin Hejazi, Vahid Damanpak, Mostafa Vahedian, Mohammadamin Sattari, Mohammadreza Seyyedmajidi

**Affiliations:** ^1^Clinical Research Development Center, Qom University of Medical Sciences, Qom, Iran; ^2^Golestan Research Center of Gastroenterology and Hepatology (GRCGH), Golestan University of Medical Sciences (GOUMS), 10th Azar, 5 Azar Boulevard, Gorgan, Golestan Province, Iran

## Abstract

*Background*. *Helicobacter pylori* (HP) infection is the most common infection in the world and coronary artery disease (CAD) is probably associated with it. The aim of this prospective study was to evaluate the association between HP infection and CAD in suspected patients referred for coronary angiography. The coronary angiography was performed using Judkins method and patients were assigned to participate in CAD positive (>50% luminal diameter stenosis) and negative groups. The serum HP IgG antibody was checked. *Results*. Positive and negative CAD groups consisted of 62 and 58 patients, respectively. HP was more prevalent among CAD+ patients, and with increasing the number of coronary arteries with stenosis, the HP seropositivity increased so that 76.3% of patients with multiple vessel diseases (MVD) and 70% of patients with single vessel diseases (SVD) were HP seropositive versus 50% in control group (*P* = 0.006). Positive CAD was significantly associated with HDL level (*P* = 0.01) and ESR level (*P* = 0.006). Also, CAD+ patients had higher CRP levels than controls and it was statistically different between SVD group and controls (*P* < 0.05). *Conclusion.* HP infection is more prevalent in CAD positive patients and, in case of proving causal relationship, it can be considered as a reversible risk factor for CAD.

## 1. Introduction

Nowadays the most common risk factors for atherosclerosis process which cause coronary heart diseases (CHD) include diabetes, dyslipidemia, hypertension, and smoking [1]. The inflammation processes and atherogenesis have many similarities, and the role of an active inflammatory process in atherosclerosis pathogenesis of the coronary circulation is growing. Significantly, monocytes and macrophages are recognized as components of atheromatous plaques for several years. The risk of cardiovascular events is associated with increased levels of the acute phase proteins, fibrinogen, C-reactive protein (CRP), and proinflammatory cytokines [2]. For this reason, chronic inflammation is considered as a risk factor for CHD, and vascular injury, inflammation, and thrombosis are considered to cause atherosclerosis whereas the stimulus that generates the inflammatory response has remained unclear [3, 4].

*Helicobacter pylori* (HP) infection is the most common infection round the world, particularly in the developing countries, and is an etiological factor for the development of peptic ulcer and gastric cancer [5]. HP infection is reported to be associated with many extra gastrointestinal manifestations such as hematological diseases like idiopathic thrombocytopenic purpura (ITP) and unexplained iron deficiency anemia; neurological disorders like stroke and Parkinson and Alzheimer's disease; obesity and skin disorders. Among these, ITP and unexplained iron deficiency anemia have the best evidence and high quality studies show the improvement of iron deficiency anemia and ITP after HP eradication [6–8].

Many reports suggest that chronic infections may be associated with atherosclerosis and vascular disease. Placing the inflammation as a cardiovascular risk factor in one hand and HP involvement in extra digestive disorders on the other hand made researches evaluate the HP role in atherosclerosis processes [9].The evidence of its association with CAD is weak and many of the results may be erroneous [6–8].

During the last years, many studies have been performed on the relationship between HP infection and atherosclerotic diseases such as stroke and ischemic heart disease [10]. Some studies have found the role of CagA positive strain of this pathogen in CHD [11, 12]. Khodaii et al. confirmed that the patients with AMI had a significantly higher prevalence of HP infection and CagA seropositivity than the control population [13].

On the contrary, some studies did not show any association between HP and CAD [14, 15]. It is assumed that an undetected chronic infection may be behind these changes in inflammatory markers and highlights microorganisms that are commonly detectable in asymptomatic individuals. Hence, the aim of this study was to evaluate the association between HP infection and coronary artery disease.

## 2. Materials and Methods

### 2.1. Patients

In this cross-sectional study, 120 patients with suspected CAD referred to Shahid Beheshti Hospital of Qom (Iran) for coronary angiography were prospectively enrolled from January to June 2013 and all of them underwent physical examinations. In data evaluation, age, gender, body mass index (BMI), patient's history (history of hypertension (indicated by systolic blood pressure ≥140 mmHg, diastolic blood pressure of ≥90 mmHg, or antihypertensive medication), diabetes mellitus, stroke, CCU admission, cardiac diseases and cardiac failure, renal insufficiency, and smoking (Patients who had stopped smoking for 10 years or less were classified as smokers)), and biochemical parameters (hemoglobin, leucocytes, thrombocytes, total cholesterol, low density lipoprotein (LDL) cholesterol, high density lipoprotein (HDL) cholesterol, triglycerides, creatinine, glycemia, CRP, and ESR) were recorded.

Patients with severe renal (creatinine >2 mg/dL) or hepatic failure, anemia, endocrine or neurological diseases or malignancies, and previous HP infection treatment were excluded.

After 8 hours of fasting overnight, venous blood samples were obtained and stored at 4°C. Serum was acquired by centrifugation of blood samples at 2000 r/min for 15 minutes, immediately after sampling. The serum HP IgG antibody was measured using ELISA kit, Padtan Elm Co., Iran, and seropositivity HP was detected based on the serum titers of higher than 30 AU/mL.

### 2.2. Coronary Angiography

Coronary angiography was performed by femoral artery using Judkins method [16]. Two experienced cardiologists unaware of the patients' enrollment reviewed all angiograms. If they did not have the same view, the third cardiologist saw the angiographic film and then, based on angiographic results, patients were divided into two groups with and without coronary artery disease.

Coronary artery disease was defined as more than 50% luminal diameter stenosis of at least one coronary artery and patients with CAD were divided into groups of single- or multi vessel disease. Patients without CAD were divided into subgroups of insignificant atherosclerosis (luminal diameter narrowing <50%) or normal (without luminal diameter narrowing).

### 2.3. Statistical Analysis

Basic statistical description of the study population adopted percentage proportions for categorical parameters while the continuous variables were expressed as mean and 95% confidence interval (CI). Significant differences among groups were tested with one-way ANOVA and Bonferroni post hoc test for continuous variables and *λ* ^2^ test for categorical variables. Bonferonni post-hoc test was adopted as a solution for multiple testing problems; it is the correction for increased level of type I error during multiple testing with objective to reach 0.05 for all tests. Predictors in logistic regression were described by their odds ratio and CI; their statistical significance was tested using Wald test which is a standard test for testing whether an independent variable has a statistically significant relationship with a dependent variable. Hosmer and Lemeshow test was used for statistical significance of the whole logistic models, analyses were performed using SPSS 16.0, and a *P* value less than 0.05 was considered as being statistically significant.

### 2.4. Ethics

All individuals signed informed consent prior to their enrolment in the study. Also, the study was planned according to the ethical guidelines following the Declaration of Helsinki and Ethics Committee of Qom University of Medical Sciences approved it.

## 3. Results

In this study 120 patients were allocated in two groups according to positive (62 patients) and negative CAD (58 patients). Detailed division in subgroups with single- and multi vessel CAD (positive CAD) and insignificant atherosclerosis group and control group (negative CAD) is presented in Tables 1-2.

The differences in basic characteristics and risk factors are presented in Table 1. It had significant differences between patients with MVD and controls by age and BMI (*P* < 0.05). Otherwise, in male gender, a significant difference was found between patients with MVD and patients with insignificant atherosclerosis (*P* < 0.05). Although patients with CAD had higher rates of hypertension and diabetes, it was not statistically significant (Table 1).

The basic differences in laboratory parameters are presented in Table 2. *Helicobacter pylori* infection had a higher prevalence in patients with coronary artery disease and the greater the number of coronary artery stenosis is, the higher the rate of HP infection was observed so that 76.3% of patients with MVD and 70% of patients with SVD had HP infection while it was 50% in normal patients and this difference was statistically significant (*P* < 0.05) (Table 2).

Patients with CAD had higher CRP levels compared to normal patients and this difference was statistically significant between SVD patients in comparison with control group (*P* < 0.05) (Table 2). Also, creatinine level was significantly decreased in both SVD and insignificant atherosclerosis group against controls (*P* < 0.05). HDL cholesterol was significantly lower in SVD group against insignificant atherosclerosis group and also it was significantly higher in insignificant atherosclerosis group against controls.

The linear logistic regression showed association of patient's biochemical markers, basic characteristics, and risk factors on presence of CAD (Table 3). From biochemical markers, positive CAD was significantly associated with HP infection (OR = 3.86, 95% CI = 1.48–10; *P* = 0.006) and HDL (OR = 0.92, 95%; CI = 0.86–0.96; *P* = 0.01) and ESR level (OR = 1.07, 95%, CI = 1.02–1.13; *P* = 0.006) (Table 3). On the other hand, presence of positive CAD was influenced only by HP infection and HDL and ESR level. From clinical factors, significant relationship was found between age, male gender, and positive CAD (SVD and MVD as well).

## 4. Discussion

It seems that CHD is one of the extra gastrointestinal diseases and some studies showed its association with HP infection [15, 17]. The role of inflammation mechanism in the pathogenesis and progression of coronary artery disease has been increasingly explored but still remains to be elucidated. Epidemiological studies based on serological findings have suggested an association between chronic HP infection and atherosclerosis, although controversies exist. In Izadi et al.'s study on 105 patients under CABG, PCR test result was positive *Helicobacter* species for 31 (29.5%) specimens from coronary artery atherosclerotic plaques. Also in serologic tests 25 (23.8%) were positive for HP immunoglobulin A (IgA) and 56 (53.3%) were positive for anti-HP immunoglobulin G (IgG) [18]. This study and other studies suggest this hypothesis that HP can be associated with CAD or even consider it as a risk factor that plays a role in atherosclerosis plaque formation [9, 19]. HP infection has been suggested to influence the development of atherosclerotic changes in coronary arteries, indicating a damaging effect of this bacterium or its products (e.g., cytokines, endotoxins, cytotoxins, and other virulence factors) on the coronary endothelium.

We found a positive association between HP infection and CAD in addition to its severity whereas 76.3% of people with MVD and 70% of patients with SVD were HP seropositive while it was 50% in control group and 52.8% in patients with nonspecific coronary artery involvement. Also, a positive correlation was found between HP seropositivity and CAD (OR = 3.86, 95% CI = 1.48–10; *P* = 0.006). This is similar to Rogha et al.'s study which found a positive association between HP seropositivity and CAD in 112 patients candidate for coronary angiography (OR 3.18, 95% CI = 1.08–9.40) [9]. Also, Vcev et al. showed this positive association [19]. The results of this study showed a higher seroprevalence of HP infection in patients with CAD compared to controls (78.8% versus 58.3%). However, in this study HP seropositivity was not associated with coronary artery disease risk factors like smoking, total cholesterol, BMI, diabetes mellitus, hypertension, and socioeconomic status either in the whole study population or in the patients and control subjects analyzed separately [19].

In our study, HP seropositive patients had different risk factors which were observed in other studies. Seropositivity rates for HP were significantly higher in patients with coronary artery disease than in controls (80.2% versus 54.5%; *P* < 0.05) [20]. Even some researchers stated that this infection will deteriorate the PTCA outcome. Kowalski found that the mean lumen loss after PTCA with stent in HP-IgG positive patients is higher compared to the HP-IgG negative patients (*P* = 0.0196) [21]. In contrast, Rogha et al. did not confirm the association between HP and CAD. They studied 105 subjects and found that HP infection and CagA Ab were not significantly higher compared to the patients with severe and mild CHD (*P* = 0.28 and *P* = 0.68, resp.). Colonization of CagA positive HP did not significantly associate with severity of CHD (OR = 1.05, 95% CI = 0.33–3.39) [9].

In Jin et al.'s study 30.7% of the normal control group and 40.6% of the CAD group were HP infection positive but there was no statistical difference [15]. Wald et al. found no association between HP seropositivity and ischemic heart disease in their study [22]. Strachan and colleagues followed the patients for 13 years and suggested that although HP does not seem to cause CAD, it increases the mortality rate in CAD patients (OR = 1.52 (95% CI, 0.99 to 2.34)) [23].

It has been suggested that HP influences the IHD development through different pathways like endothelial cells colonization, lipid profile changes, increased coagulation and platelet aggregation levels, induction of molecular mimicry mechanisms, and the promotion of a low-grade systemic inflammation [10].

On the other side, chronic HP infection is known to increase the pH level of the gastric juice and to decrease ascorbic acid levels, both of which will cause folate absorption reduction. Low folate hampers the methionine synthase reaction. This will increase blood hemocysteine concentration which results in damage of endothelial cells [24]. Siddiqui et al. evaluated the hemocysteine level in CAD patients with HP infection and found no difference [25]. Also, large population-based studies suggested the relation between the CRP levels and risk of coronary artery disease [24]. The association between HP infection and plasma levels of CRP, has been investigated by Pienjzek et al. [26].

In our study, like Tamer et al. and Siddiqui et al.'s study, the CRP level was higher in CAD patients. In Tamer et al.'s study, CRP level was different among patients with and without CAD but HP infection did not differ in both groups [20].

Although CAD patients had higher CRP and ESR levels in our study, but assigning them into HP+ve and HP−ve groups, no difference was detected. Gastric infection with HP may also induce the synthesis of acute phase reactants and activate immune mechanisms due to cross-reacting antibodies to HP and heat shock protein (HSP 60/65) with endothelial-derived HSP 60/65 [27, 28]. Mendall et al. found a strict correlation between the increment in serum levels of some proinflammatory cytokines (IL-1b, IL-8, and TNF-*α*) and cardiovascular risk factors [27].

Also some studies suggest the coagulability stimulation caused by HP. Niemelä et al. and de Luis et al. have demonstrated that HP infection causes thrombotic protein changes [29, 30]. The association between HP infection and fibrinogen has been investigated by Pienjzek et al. [26]. One interesting hypothesis is stimulated platelet aggregation by *Helicobacter pylori*. Results showed that some HP strains are able to bind to the von Willebrand factor to interact with glycoprotein Ib and to induce platelet aggregation in humans and HP may eventually affect IHD by eliciting thrombosis [31]. Also, Elizalde shows that circulating platelet aggregates and activated platelets were also detected in HP infected patients [32].

Thromboxane (TXB) is an index of platelet activation which was found to be significantly higher in the HP+ and CAD+ (*P* = 0.05), but not in the HP+ CAD− (*P* = 0.88) when compared with HP− CAD− [33]. HP infection was reported by Pellicano et al. to induce also platelet activation and aggregation as well as an increase of plasma levels of triglycerides and various proatherogenic factors including *hemocysteine* [34]. Also, the seroprevalence of CagA positive strains was significantly higher in patients with acute MI and UA than controls (86.7, 91.7, and 58.3%, resp.) [17]. Kowalski also reported that, in homogenous group of patients, those with CAD had significantly higher HP IgG and CagA seroprevalence (69.79% versus 58.20%) as compared to non-CAD controls (40.62% versus 35.89%) [21].

Although some studies do not confirm it, colonization of CagA positive HP is not an independent risk factor for severe coronary heart disease [9]. Some researchers evaluated the effect of HP through metabolic changes (lipid profile). Rahman et al. compared patients suffering from HP and CAD with those suffering from HP without CAD and analyses of lipid profiles showed that while triglycerides, total cholesterol, and LDL had no significant changes, HDL was significantly lower in both groups [33]. HP specific IgG was positively correlated with triglyceride level [35]. Niemelä et al. and de Luis et al. have shown that HP infection causes various lipids and there is a good correlation between HP infection and decreased HDL cholesterol [29, 30]. Also, Jia et al. showed that HDL-c was significantly decreased in patients with HP infection, indicating that HP infection may result in decreased blood HDL-c levels, which then contributes to the development of coronary atherosclerosis [36]. But in our study, lipid profile was not different between seropositive and seronegative patients and colonization of HP is an independent risk factor for coronary heart disease.

## 5. Conclusion

According to our study, HP seropositive patients are at higher risk for CAD and the number of their involved arteries is greater. Given the high prevalence of HP infection and as coronary artery disease is the major cause of mortality in this population, this issue is of importance and in case of proving this causal relationship, we can avoid mortality due to CAD. Also, it seems that, in PTCA patients, HP treatment reduces the stenosis.

## Figures and Tables

**Table 1:** Description of patients with comparison of CAD (basic characteristics and risk factors).

	All patients (*N* = 120)	MVD (*N* = 36)	SVD (*N* = 26)	Insignificant atherosclerosis (*N* = 38)	Control group (*N* = 20)
Basic characteristics					
Age^†^	56.2 (54.14–58.26)	59.4 (55.7–63.1)*	56.4 (52.3–60.5)	56.3 (52.5–59.9)	49.9 (43.7–56.1)
Male gender^†^	50.8	66.7^#^	46.2	39.5	50
Height (cm)	162.1 (160.4–163.8)	164.1 (161.1–167.1)	162.1 (157.5–166.7)	160.1 (157.4–162.8)	162.2 (158.1–166.3)
Weight (kg)	75.4 (72.8–78)	74 (69.5–78.5)	73 (67.1–78.9)	74.3 (69.8–78.8)	82.7 (74.6–90.8)
BMI (kg/m^2^)^†^	28.7 (27.8–29.6)	27.5 (25.9–29.1)*	27.7 (25.8–29.6)	29 (27.3–30.7)	31.6 (28.6–34.6)

Risk factors (%)					
Hypertension	60	61.1	73.1	57.9	45
Diabetes mellitus	29.2	36.1	26.9	26.3	25
History of cardiac disease^†§^	30.8	38.9^#^	42.3^#^	15.8	30
History of CCU admission^††§^	45.8	55.6^##^	61.5^##^	23.7**	50
History of cardiac failure	50.8	50	61.5	42.1	55
Smoking experience	17.5	27.8	11.5	13.2	15

Categorical variables are presented in %, continuous variables as mean (95% confidence interval).

^†^Significant difference among groups: ANOVA/Chi-square test (*P* < 0.05), ^††^significant difference among groups: ANOVA/Chi-square test (*P* < 0.001), *significant difference versus control group: Bonferroni test (*P* < 0.05), **significant difference versus control group: Chi-square test (*P* < 0.001), ^§^significant difference between CAD pos. and CAD neg.: Chi-square test (*P* < 0.050), ^#^significant difference versus insignificant atherosclerosis: Chi-square test (*P* < 0.05), ^##^significant difference versus insignificant atherosclerosis: Chi-square test (*P* < 0.001); BMI: body mass index.

**Table 2:** Laboratory markers in groups of CAD.

	All patients (*N* = 120)	CAD pos.		CAD neg.	
		MVD (*N* = 36)	SVD (*N* = 26)	Insignificant atherosclerosis (*N* = 38)	Control group (*N* = 20)
Laboratory markers					
HP Seropositive (%)^†§^	62.5	76.3^#¥^	70^#^	52.8	50
CRP^†^	6.25 (4.55–7.95)	6.2 (2–10.2)	8.4* (3.8–13.1)	5.2 (3.7–6.7)	5.5 (2.4–8.6)
ESR^†§^	13.7 (11.1–16.3)	17.2* (10.8–23.5)	17* (10.7–23.3)	10.3 (7.1–13.5)	9.5 (5.2–13.8)
Total Chol	145 (138–152)	155.5 (142.5–168.5)	136.5 (122.3–150.7)	144.2 (132.6–155.8)	138.8 (120–157.8)
HDL^††^	48.5 (46.6–50.4)	49.5 (45.9–52.9)	43.8 (39.1–48.5)^##^	53.6 (50.5–56.7)**	43.2 (39.9–46.5)
TG	149.7 (133.3–166.1)	154.6 (123–186.2)	150.7 (120.9–180.3)	140.9 (111.3–170.5)	156 (104.5–208.1)
LDL	69.6 (63.1–76.1)	79.3 (66.1–92.5)	61.8 (49.7–73.9)	68.6 (55.2–82)	64 (52–76)
Creatinine^†^	1.08 (0.95–1.2)	1.06 (0.99–1.2)	0.94 (0.89–1)*	0.95 (0.9–1)*	1.5 (0.76–2.2)

Categorical variables are presented in %, continuous variables as mean (95% confidence interval), and due to not normal distribution logarithmic transformation of the data was needed.^†^significant difference among groups: ANOVA/Chi-square test (*P* < 0.050), ^††^significant difference among groups: ANOVA/Chi-square test (*P* < 0.001), ^§^significant difference between CAD pos. and CAD neg.: Chi-square test (*P* < 0.050), ^#^significant difference versus insignificant atherosclerosis: Chi-square test (*P* < 0.050), ^##^significant difference versus insignificant atherosclerosis: Bonferroni test (*P* < 0.001), *significant difference versus control group: Bonferroni test (*P* < 0.05), **significant difference versus control group: Bonferroni test (*P* < 0.001), and ^**¥**^significant difference versus control group: Chi-square test (*P* < 0.050).

**Table 3:** Influence of patient's basic characteristics and biochemical parameters on presence of disease based on linear logistic regression.

Variable	OR	95% confidence interval	*P* value
Age	1.04	1.00–1.08	0.049
Male gender	3.03	1.02–8.95	0.045
HDL	0.92	0.86–0.98	0.01
ESR	1.07	1.02–1.13	0.006
HP seropositive	3.86	1.48–10	0.006
